# Prevention of Skin Damage Using Disposable Retractors in Open Hepatobiliary Surgery: A Nonrandomized Controlled Trial Comparing Film Drapes and Sewn Gauze

**DOI:** 10.7759/cureus.77116

**Published:** 2025-01-07

**Authors:** Reika Tachibana, Kentaro Hara, Amane Kitasato, Tamotsu Kuroki

**Affiliations:** 1 Department of Operation Center, National Hospital Organization Nagasaki Medical Center, Omura, JPN; 2 Healthcare Management Research Center, Chiba University Hospital, Chiba, JPN; 3 Department of Surgery, National Hospital Organization Nagasaki Medical Center, Omura, JPN

**Keywords:** disposable retractor, film draping, open abdominal hepatobiliary-pancreatic surgery, sewn gauze pad, skin injury

## Abstract

Aim

This study aimed to compare the effectiveness of film drapes and sewn gauze in preventing skin injuries caused by the use of disposable retractors during open hepatobiliary pancreatic surgery under general anesthesia.

Design

This was a single-center, nonrandomized, controlled trial.

Methods

This study was conducted at a medical center between August 2021 and May 2022. All participants were aged 20 years or older and underwent open abdominal hepatobiliary-pancreatic surgery under general anesthesia using disposable retractors.

Results

During the study period, 57 patients were enrolled: 29 in the film drape group and 28 in the sewn gauze group. Notably, the film drape group experienced 12 incidents of skin injury, whereas the sewn gauze group experienced statistically significantly fewer, at only four incidents.

Conclusions

In open abdominal hepatobiliary-pancreatic surgery under general anesthesia, the use of sutured gauze to provide moisture and pressure relief during the use of disposable retractors significantly reduces the incidence of postoperative skin injuries.

## Introduction

In many open hepato-pancreato-biliary (HPB) surgeries under general anesthesia, disposable retractors (hereafter referred to as retractors) are commonly used to maintain the surgical field. Retractors are tubular devices that use ring tension to keep a surgical site open. At Hospital A's surgical center, from January to June 2021, five of 32 open HPB surgeries (15.6%) reported medical device-related pressure injuries (MDRPI), such as epidermal detachment and blisters, occurring at the skin-contact site of the retractor ring.

The National Pressure Injury Advisory Panel defines MDRPI as injuries resulting from the use of devices designed and applied for diagnostic or therapeutic purposes. These injuries typically conform to the pattern or shape of the device and progress similarly to other types of pressure ulcers [1]. Pressure ulcers are localized injuries to the skin or underlying soft tissue, usually occurring over bony prominences. Factors contributing to pressure ulcers are classified as external factors (e.g., skin moisture, friction, and shear) and internal factors (e.g., malnutrition, age-related skin changes, and systemic functional decline) [1,2].

We explored preventive methods to address the external factors associated with retractor use. However, when we conducted a literature review, we found no reports on strategies for preventing skin injuries related to retractors. Therefore, we collaborated with operating room nurses and surgeons to propose two preventive measures. First, we suggested applying a film drape to the abdominal area where the retractor is placed. This method aimed to reduce external forces caused by friction and shear at the retractor contact site. Second, we proposed placing a sewn gauze pad between the retractor ring and the skin to prevent direct contact. The use of a sewn gauze pad was expected to prevent skin infiltration and reduce shear forces at the contact site.

In this study, we thus compared these two methods and identified an effective strategy for preventing skin injuries associated with retractor use. We anticipated that the findings of this study would contribute to the prevention of perioperative skin injuries caused by retractors, thereby reducing the incidence of secondary complications, and could form a basis for evidence-based guidelines for preventing skin injury in surgical settings.

## Materials and methods

Study design and ethical considerations

This single-center, nonrandomized, comparative study was conducted at a medical center. This study was approved by the Medical Center Ethics Review Committee (approval no. 2021097) on October 4, 2021. The study protocol registry is UMIN000056337. The study was explained in detail to all patients and their legal guardians, and written informed consent was obtained from all patients. The study was conducted and reported according to the Strengthening the Reporting of Observational Studies in Epidemiology (STROBE) guidelines [3].

The researchers provided the participants with a consent form approved by the institutional head. They explained the study in writing and orally, giving participants sufficient time to ask questions and decide whether to participate. Written informed consent was obtained after confirming that the participants fully understood the study and agreed to participate of their own free will.

Study setting and sampling

This study was conducted at a medical center between October 31, 2021, and September 30, 2022. The patients were alternately assigned to either the film drape group or the sewn gauze group. The subjects were patients aged 20 years or older who were scheduled to undergo open hepatobiliary pancreatic malignancy surgery under general anesthesia. The exclusion criteria consisted of patients with significant comorbidities that could independently affect skin integrity and patients undergoing emergency surgeries. To minimize bias due to differences in the overall health status, emergency surgeries were excluded. The procedures included in this study were hepatic regional resection, partial hepatic resection, pancreaticoduodenectomy, and distal pancreatectomy. The sample size was calculated using G*Power 3.1 (Franz Faul, Universität, Germany) with an effect size of 0.3, alpha level of 0.05, and power of 0.8. Accordingly, 57 cases were targeted.

Outcome and data collection

The primary endpoint was the number of postoperative skin injuries at the retractor site. The skin injuries were classified according to the National Pressure Ulcer Advisory Panel classification [4]. The secondary endpoint was the presence or absence of pain at the retractor-contact site in patients with skin lesions, as assessed using a numeric rating scale (NRS). The NRS scores were categorized as follows: 0, no pain; 1-3, mild pain; 4-6, moderate pain; and 7-10, severe pain [5].

Preoperative clinical findings (age, sex, height, weight, body mass index, medical history, total protein, and albumin), intraoperative factors (operation time, blood loss, retractor application time, presence or absence of retractor offloading, fluid volume, and urine volume), and postoperative factors (postoperative skin injury at the retractor site and presence or absence of pain at the retractor contact site in patients with skin lesions) were collected from the study participants. Postoperative reactive hyperemia was included if it persisted for >10 min after the retractor was removed.

Equipment Used

**Figure 1:**
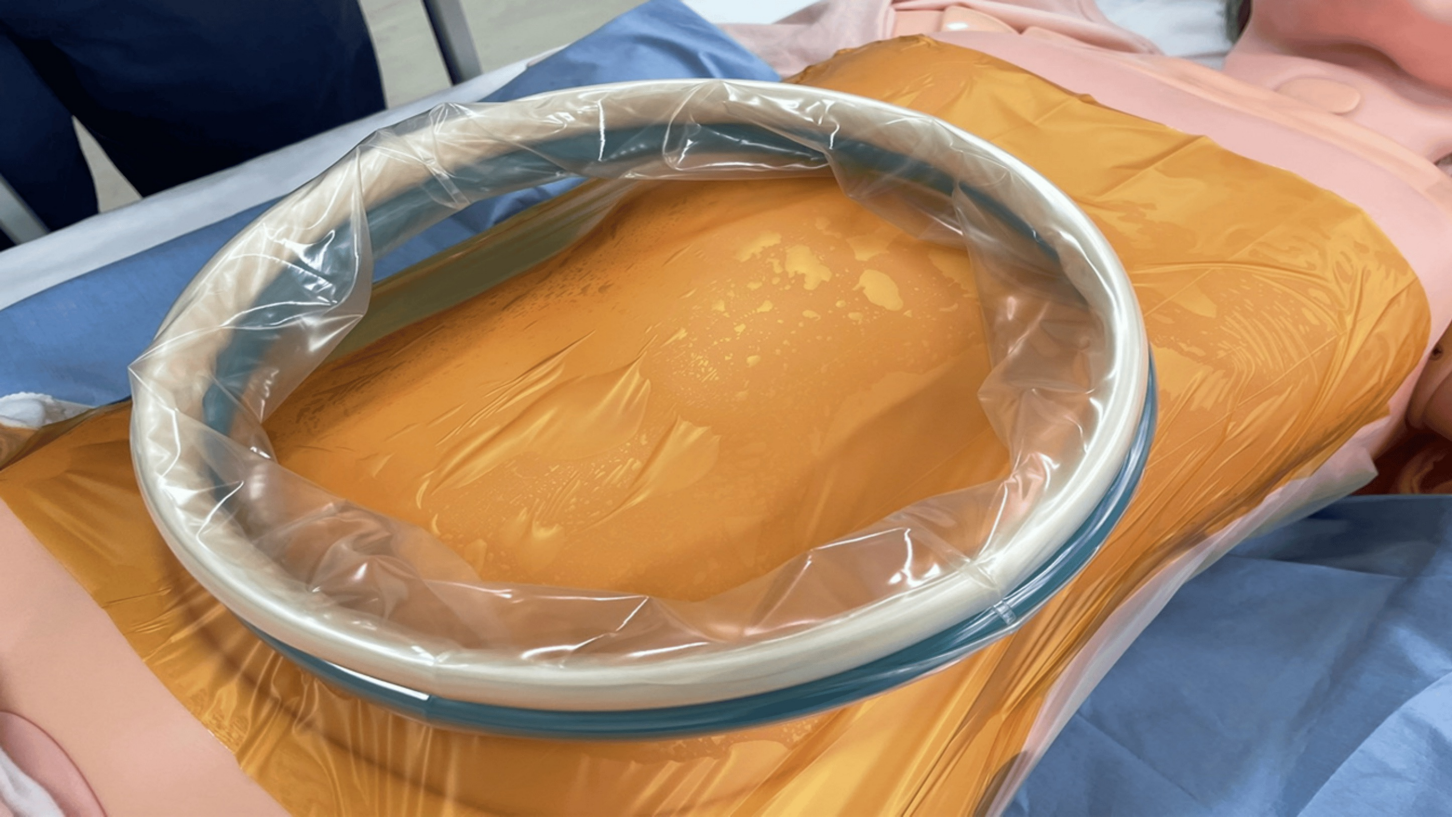
Method using film drape material Apply the film drape to the abdominal area where the retractor is placed

**Figure 2:**
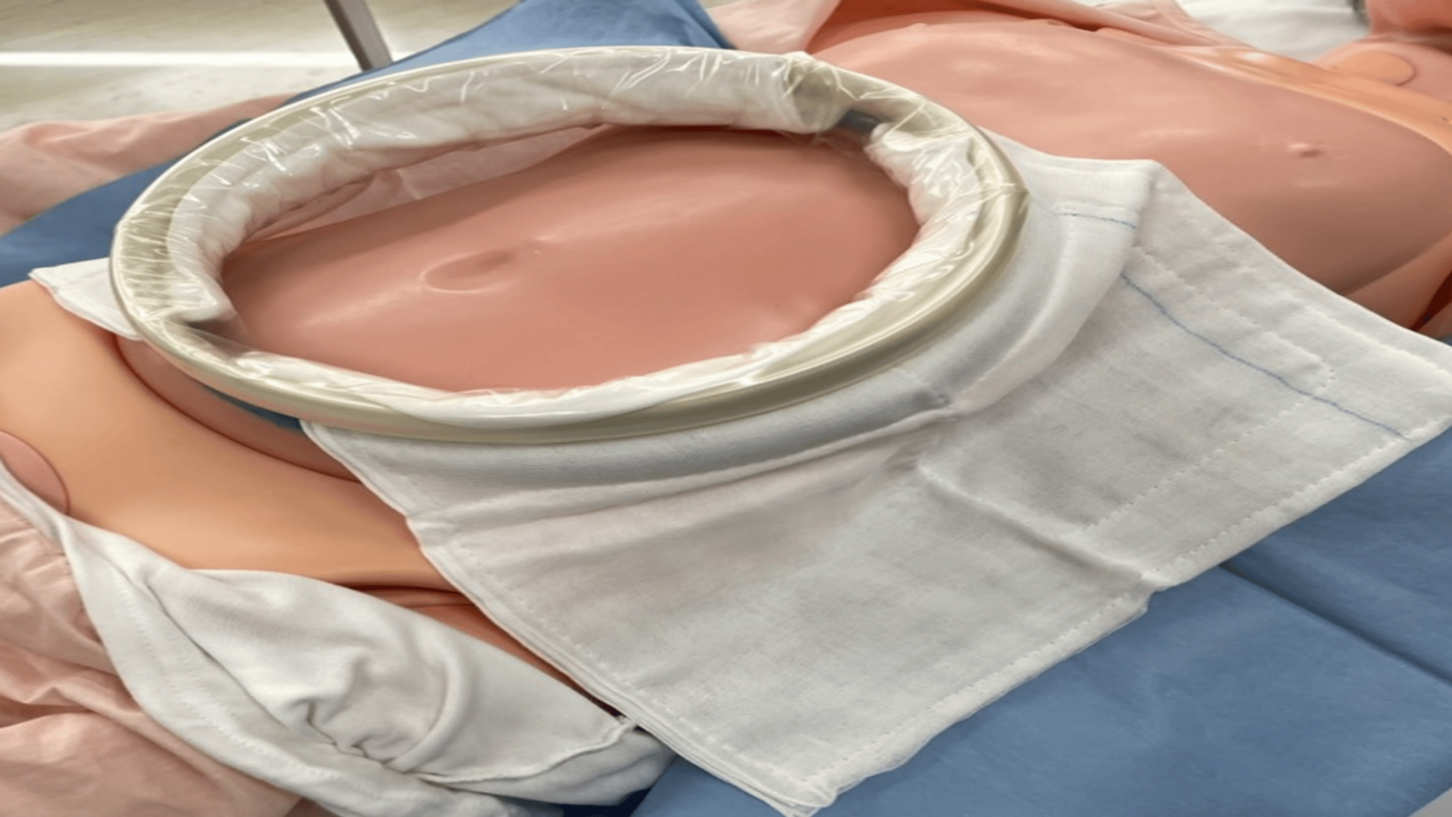
Method using sewn gauze Place the sewn gauze between the retractor ring and the skin

In surgeries, the Alexis®O Wound Protector/Retractor XL (Applied Medical, Rancho Santa Margarita, CA, USA) was used. An Ioban™2 6640EZ® 35 × 35 cm film drape was used in the film drape group (3M, St. Paul, MN, USA) (Figure 1), while a sterilized sewn handkerchief® 30 × 30 cm (Daiwa Factory Co., Ltd., Yao City, Japan) was used in the sewn gauze group (Figure 2).

Film drape: The film drape is a nonabsorbent protective material applied directly to the skin where the retractor makes contact. Its smooth surface minimizes friction and shear forces, reducing the likelihood of skin injury. However, since it does not absorb moisture, prolonged exposure to a moist environment may weaken the skin's barrier function, potentially increasing the risk of skin damage.

Sewn gauze: The sewn gauze pad, placed between the retractor ring and the skin, is designed to absorb blood and bodily fluids during surgery. This absorption helps to mitigate the moist environment around the retractor contact site, which could otherwise contribute to skin damage. Additionally, the thickness of the sewn gauze provides cushioning, reducing direct pressure on the skin, which is particularly beneficial in preventing skin injuries during prolonged surgeries.

Statistical analysis

Descriptive statistics for patient, surgical, and anesthetic information were calculated. Data are presented as means (standard deviations). Group comparisons for the primary endpoint were performed using the chi-squared (χ²) test, with a significance level of 5%. For secondary endpoints, patient background, intraoperative factors, and postoperative factors, appropriate statistical tests (χ² or Mann-Whitney U test) were used, according to the characteristics of the variables, with a significance level of 5%. Outliers were defined as values below the first quartile minus 1.5 times the interquartile range (IQR) or above the third quartile plus 1.5 times the IQR. Statistical analyses were conducted using JMP® 16 software (SAS Institute Inc., Cary, NC, USA).

## Results

Participant demographics

**Figure 3:**
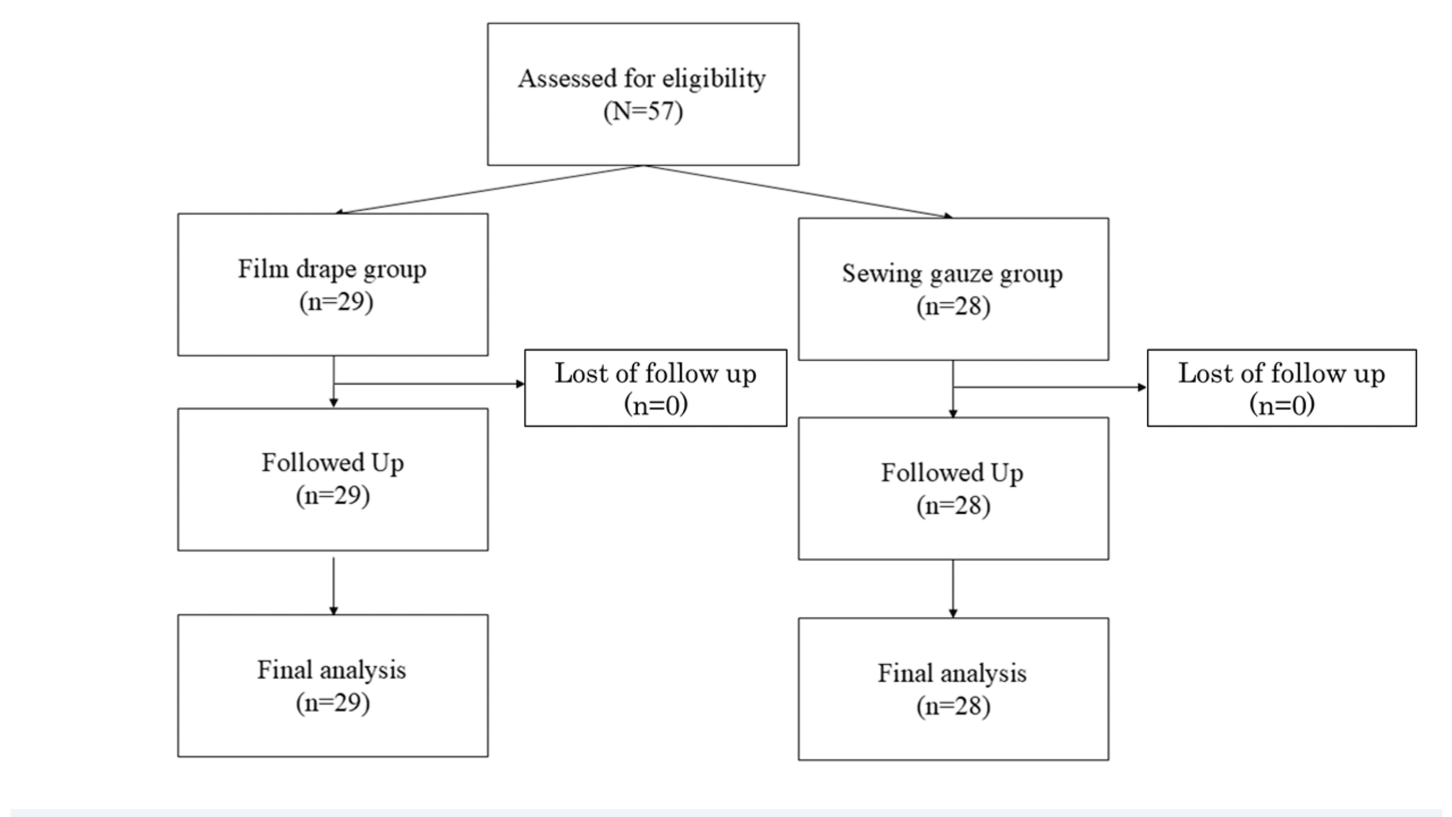
Trial STROBE diagram STROBE: Strengthening the Reporting of Observational Studies in Epidemiology During the study period, 57 patients participated, and since no emergency surgeries were involved, all 57 patients were included in the analysis

Fifty-seven patients participated in the study, with no emergency surgeries conducted; thus, data from all 57 patients (film drape group: 29 cases; sewn gauze group: 28 cases) were included in the analysis (Figure 3).

The demographic characteristics of the participants are presented in Table 1. No significant differences in patient background characteristics were observed between the two groups.

**Table 1:** Patient characteristics Note: Values are presented as median (interquartile range) or number (percentage) ^a^Mann-Whitney U test; ^b^Chi-square test; *p < .05

	Film drape group (n = 29)	Sewn gauze group (n = 28)	p-value
Age (years)^a^	72.0 (69.0-77.0)	74 (64.2-79.0)	.65
Sex^b^			.48
Male	16 (55.1%)	18 (64.2%)	
Female	13 (44.8%)	10 (35.7%)	
Height (cm)^b^	160 (151.5-166.2)	161.5 (153.6-169.6)	.30
Weight (kg)^b^	56.6 (50.15-62.45)	62.2 (51.5-71.7)	.20
Body mass index (kg/m^2^)^b^	22 (19.2-25.4)	23.3 (21.8-25.6)	.38
Preoperative total protein (g/dl)^b^	6.8 (6.55-.3)	6.8 (6.5-7.2)	.99
Preoperative albumin (g/dl)^b^	3.9(3.45-4.05)	4.0 (3.7-4.2)	.11
Diabetes mellitus^b^			.69
Yes	13 (44.8%)	14 (50%)	
No	16 (55.1%)	14 (50%)	
Steroid^b^			.24
Yes	1 (3.4%)	0 (0%)	
No	28 (96.5%)	28 (100%)	

The surgical and anesthetic factors are presented in Table 2. The surgical time was significantly shorter in the film drape group (p = .04).

**Table 2:** Surgical and anesthetic factors Note: Values are presented as median (interquartile range) Mann-Whitney U test *p < .05

	Film drape group (n = 29)	Sewn gauze group (n = 28)	p-value
Operative time (min)	266 (229-347)	305.5 (272-400)	.04*
Wearing time (min)	225 (178.5-291.5)	240.5 (219.7-320.7)	.32
Total fluid volume (ml)	2841 (2638-3776)	3153.5 (2499-4463)	.59
Urine volume (ml)	225 (135-442.5)	315 (205-587.5)	.22
Amount of bleeding (ml)	455 (185-765)	505 (240-807.5)	.54
Type of Incisions			.06
Vertical incisions	20	14	
Oblique incisions	3	10	
Mercedes incisions	6	4	

Postoperative factors are compared in Table 3. Postoperative skin damage at the retractor-contact site occurred in 12 cases (41.3%) of the film drape group and four cases (14.2%) of the sewn gauze group (p = .02). None of the patients with retractor-related skin damage experienced postoperative pain.

**Table 3:** Postoperative factors Note: Values are presented as median (interquartile range) or number (percentage) Chi-square test *p < .05

	Film drape group (n = 29)	Sewn gauze group (n = 28)	p-value
Postoperative pressure injury			.02*
Yes	12 (41.3%)	4 (14.2%)	
No	17 (58.6%)	24 (85.7%)	
Postoperative pain			-
Yes	0	0	
No	29 (100%)	28 (100%)	

## Discussion

In this study, no significant difference was observed in the patient backgrounds between the film drape group and the sewn gauze group. This result suggests that the patient backgrounds were appropriately matched between the two groups, and there was no bias due to background factors in the comparison of the preventive methods.

We hypothesized that the primary factors contributing to the occurrence of MDRPI with retractor use are friction, shear stress, pressure, bleeding, and the resulting moist environment at the contact site. Therefore, we considered that using either film draping or sewn gauze would reduce external forces and prevent skin damage, potentially yielding effective results with both methods. However, the results of this study showed that compared to the film drape group, the sewn gauze group had significantly reduced postoperative skin damage.

This difference is likely due to the difference in the constituting materials between the film draping and the sewn gauze. With film draping, we anticipated a "sliding effect" against friction and shear forces. Previous research using film dressings for pressure ulcer prevention in patients undergoing supine surgery showed that the incidence of postoperative skin damage was significantly lower in the group with than in the group without film dressings [6]. This suggested that reducing the friction and shear forces is important for preventing skin damage. However, in the present study, the use of sewn gauze resulted in a significantly lower incidence of skin damage. This may be due to the "thickness" of sewn gauze as compared to that of film draping. Although film draping is expected to prevent friction and shear, its protection against direct pressure is insufficient.

The second contributing factor is the pressure exerted locally on the skin. Disposable retractors exert pressure on the wound site during prolonged surgery due to their vinyl and frame components. Therefore, we hypothesized that using a sewing gauze helps avoid direct pressure on the retractor site, thereby contributing to skin damage prevention. Skin damage is more likely to occur in patients with malnutrition, diabetes, or liver disease; however, even in patients without such risks, circulatory dysfunction occurs when capillary pressure exceeds 32 mmHg, and irreversible changes occur when it exceeds 70 mmHg for more than two hours [7,2]. No significant difference was found in the nutritional status between the two groups in this study.

Third, the management of the wet environment was considered relevant. The sewn gauze served to reduce the wet environment to some extent by absorbing intraoperative blood and body fluids. However, we also recognize that the absorbed blood and body fluids could accumulate, and the sewn gauze itself could become wet. Therefore, while the use of sewn gauze did not completely eliminate the wet environment, it was considered beneficial in terms of preventing fluid retention directly on the skin surface. On the other hand, film drapes do not absorb moisture, so there was a high likelihood that fluid would spread to the skin surface and a wet environment would persist. It has been reported that a persistent moist environment causes the stratum corneum to swell and the skin barrier function to deteriorate [8-10]. In this study, the use of sewn gauze reduced the wet environment during retractor use to a certain degree and may have contributed to the maintenance of skin barrier function. In addition, even when the operation time was long, the sewn gauze group was more effective in preventing skin damage, suggesting that the management of a moist environment is important for skin protection.

A secondary benefit of using a sewing gauze is its cost-effectiveness. The cost of the film drape used in the A hospital's surgical center is ¥780 per sheet, whereas sewn gauze costs only ¥150 per sheet. Therefore, sewn gauze is a low-cost yet highly effective option for preventing skin damage during retractor use. In Japan, preventive dressings are not covered by insurance. However, preventing skin damage during invasive surgeries, such as open abdominal surgeries for liver, bile duct, and pancreatic cancers, is crucial to facilitate postoperative recovery and early discharge. Based on these findings, sewn gauze is recommended for preventing skin damage associated with the use of disposable retractors.

The limitations of this study include being a single-center prospective observational study with a small sample size. To obtain statistically reliable results, future studies should include cases from multiple centers. Furthermore, to clarify the relationship between retractor use and skin damage, the patient's skin condition should be observed preoperatively. Additionally, the type of incision was not standardized in this study; therefore, future studies should standardize the incision method. This would help eliminate biases related to the surgical technique and yield more reliable results.

## Conclusions

Considering the mechanisms of skin damage associated with retractor use, we demonstrated that sewn gauze, which is specialized for managing pressure and moisture, is more effective than film draping in preventing skin injuries during retractor use. Additionally, sewn gauze provides an efficient solution for preventing skin damage during surgeries involving retractor use at a lower cost. Overall, we recommend the use of sewn gauze as an effective material for preventing skin damage during the use of disposable retractors, particularly in open abdominal liver, gallbladder, and pancreatic surgeries.
